# Ligand regulation of a constitutively dimeric EGF receptor

**DOI:** 10.1038/ncomms8380

**Published:** 2015-06-10

**Authors:** Daniel M. Freed, Diego Alvarado, Mark A. Lemmon

**Affiliations:** 1 Department of Biochemistry and Biophysics, University of Pennsylvania Perelman School of Medicine, 322A Clinical Research Building, 415 Curie Boulevard, Philadelphia, Pennsylvania 19104-6059, USA

## Abstract

Ligand-induced receptor dimerization has traditionally been viewed as the key event in transmembrane signalling by epidermal growth factor receptors (EGFRs). Here we show that the *Caenorhabditis elegans* EGFR orthologue LET-23 is constitutively dimeric, yet responds to its ligand LIN-3 without changing oligomerization state. SAXS and mutational analyses further reveal that the preformed dimer of the LET-23 extracellular region is mediated by its domain II dimerization arm and resembles other EGFR extracellular dimers seen in structural studies. Binding of LIN-3 induces only minor structural rearrangements in the LET-23 dimer to promote signalling. Our results therefore argue that EGFR can be regulated by allosteric changes within an existing receptor dimer—resembling signalling by insulin receptor family members, which share similar extracellular domain compositions but form covalent dimers.

## Abstract (web summary)

Whereas epidermal growth factor-induced dimerization is considered essential for EGFR signalling, the structurally related insulin receptor is a disulfide-linked dimer. Here the authors show that *C. elegans* EGFR is constitutively dimeric and undergoes subtle structural changes upon ligand binding that likely underlie allosteric activation.

## Graphical abstract

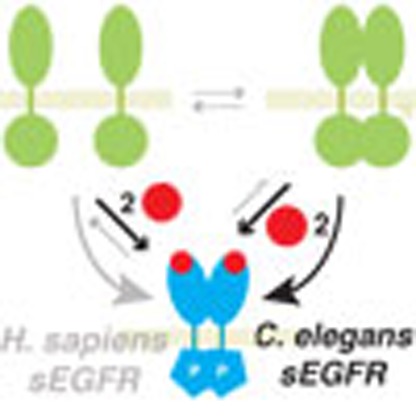

Receptor tyrosine kinases (RTKs) control many cellular processes and play causative roles in diseases such as cancer, where they are important therapeutic targets^1^. Early work with the epidermal growth factor (EGF) and platelet-derived growth factor (PDGF) receptors established that these RTKs signal as dimers, and further suggested that signalling requires ligand-induced receptor dimerization^2^,^3^. Several biochemical and structural studies of EGF receptor (EGFR) have subsequently argued that the extra- and intracellular regions of the receptor are structurally independent and flexibly linked^4^,^5^,^6^,^7^—consistent with models in which dimerization itself (induced by ligand binding) is the key event in receptor activation. On the other hand, reports that many RTKs can dimerize without ligand (forming inactive ‘preformed dimers’)^8^ argue that the signalling mechanism cannot be this simple—as does the fact that RTKs from the insulin receptor (IR) family are covalently linked dimers^9^. Structural analysis of IR family members has provided valuable insight into how these constitutively dimeric RTKs are regulated by their ligands^10^,^11^,^12^,^13^, but whether (and how) RTKs that are not disulfide-linked dimers are also allosterically regulated through analogous mechanisms remains unclear. Indeed, the precise nature (or oligomerization state) of a ‘preformed RTK dimer’ has not been described outside the IR family.

In studying EGFR orthologues from different phyla, we discovered that the isolated extracellular region of the *C. elegans* EGFR^14^, called LET-23, dimerizes strongly—with a sub-micromolar dissociation constant (*K*_d_^dim^)—regardless of the presence of its EGF-like ligand (LIN-3 (ref. 15)). By contrast, the unliganded extracellular region of human EGFR is exclusively monomeric in solution (*K*_d_^dim^>1 mM^16^) and that from *Drosophila melanogaster* EGFR is predominantly monomeric (*K*_d_^dim^∼40 μM^17^)—despite the fact that the three extracellular regions share 29%–39% pairwise sequence identity (Supplementary Fig. 1). LET-23 therefore provides a unique opportunity to study the extracellular region of a preformed EGFR dimer. Using biophysical and biochemical approaches, we show that ligand-induced changes in this preformed dimer are relatively small in scale, indicating that EGFR signalling can be regulated by relatively subtle allosteric changes within a receptor dimer. These findings in turn suggest mechanistic similarities between EGFR and the constitutively dimeric IR-family receptors, which share the same domain types over much of their extracellular regions (Fig. 1a).

## Results

### The LET-23 extracellular region is constitutively dimeric

**Figure 1:**
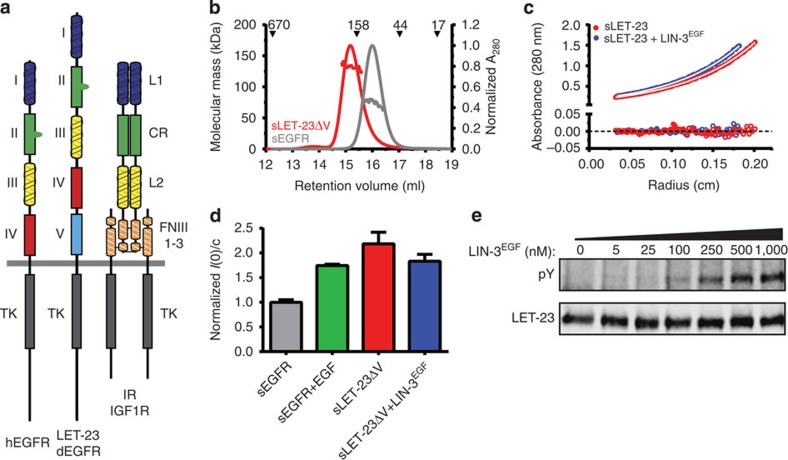
LET-23 is a ligand-regulated dimer. (**a**) Domain compositions of human EGFR (hEGFR), *C. elegans* LET-23 and *D. melanogaster* EGFR (dEGFR), and members of the covalently dimerized IR family. Domains I and III (L1 and L2 in IR) are β-helix/solenoid domains. Domains II, IV and V (CR in IR) are cysteine rich. IR also has fibronectin type III (FNIII) domains. Intracellular tyrosine kinase (TK) domains are marked. Note the dimerization arm emanating from domain II in the EGFRs. (**b**) Size-exclusion chromatography–MALS (SEC-MALS) data for sLET-23ΔV (red) and human sEGFR (grey) at eluted concentrations of ∼1 μM. Circles denote molecular masses determined by in-line MALS (left axis). Including glycosylation, the monomeric molecular masses of sEGFR and sLET-23ΔV are both ∼80 kDa—with sEGFR running as a monomer and sLET-23ΔV as dimer. Retention volumes for protein molecular mass standards are marked (with kDa values) across the top of the figure. (**c**) Representative SE-AUC data for sLET-23 (8 μM, 8,200 r.p.m.). Without ligand (red circles), the data across all repeats fit well to a single ideal 190±22 kDa species—approximately twice the monomeric mass (∼91 kDa). In the presence of 60 μM LIN-3^EGF^ (blue circles), the fit is essentially unchanged, yielding a molecular mass of 185±6 kDa across all repeats (see Table 1). Fits to the data are shown as white curves superimposed on the data points and residuals are shown below the fits as open circles. (**d**) SAXS-derived normalized *I*(0)/c values for sEGFR and sLET-23ΔV, with and without bound ligand (added at 61–110 μM) at pH 8, using home-source data. The average *I*(0)/c for unliganded (monomeric) sEGFR was set to a relative value of 1.0 and all other *I*(0)/c values were normalized to this value, with mean±s.d. presented for 3–14 independent measurements. (**e**) Western blotting showing LIN-3^EGF^-induced phosphorylation of full-length LET-23 in *D. melanogaster* S2 cells. The uncropped gel and molecular weight markers are shown in Supplementary Fig. 2.

During initial purification, we found that—unlike other EGFR extracellular regions^16^,^18^—the unliganded LET-23 extracellular region (sLET-23) migrates solely as a dimer in size-exclusion chromatography, and that this behaviour is retained when the invertebrate-specific domain V (Fig. 1a) is deleted to yield sLET-23ΔV (Fig. 1b). Unliganded sLET-23ΔV runs as an ∼140 kDa dimer (compared with a predicted monomeric mass of 71 kDa)—as also confirmed by in-line multi-angle light scattering (MALS). By contrast, Fig. 1b shows that the unliganded human EGFR extracellular region (sEGFR) runs true to its monomeric molecular mass (∼80 kDa). Strong dimerization of sLET-23 (including domain V) was also evident in sedimentation equilibrium analytical ultracentrifugation (SE-AUC) studies (Fig. 1c), which detected a single species with a molecular mass of 190±22 kDa (compared with the expected monomer mass of 91 kDa). Importantly, SE-AUC studies revealed that adding excess ligand (LIN-3^EGF^: the purified EGF domain from LIN-3, added at 60 μM) did not alter the oligomerization state of sLET-23 (Fig. 1c and Table 1), despite binding with an affinity that ensures saturation of the receptor under the conditions of this experiment (*K*_d_=2–4 μM; Supplementary Fig. 2a,b). Small-angle X-ray scattering (SAXS) studies gave the same result (Fig. 1d and Supplementary Fig. 4). SAXS provides a shape-independent measure of the weight-averaged molecular mass of the scattering species through the parameter *I*(0)/c, which is the concentration-normalized scattering intensity extrapolated to zero angle. For sEGFR, *I*(0)/c nearly doubles on addition of EGF (Fig. 1d), as we have reported previously^16^. As unliganded sLET-23ΔV is dimeric, its measured *I*(0)/c value is approximately twice that of unliganded human sEGFR and approximates *I*(0)/c for the EGF-induced sEGFR dimer. Importantly, adding excess LIN-3^EGF^ does not increase *I*(0)/c for sLET-23 (Fig. 1d, see also Supplementary Fig. 4). Taken together, these results reveal that the isolated LET-23 extracellular region exists as a dimer, with no change in oligomerization state on association with its activating ligand.

### LIN-3 activates dimeric LET-23 in cells

Our biophysical studies show that sLET-23 is entirely dimeric at concentrations as low as 1 μM, arguing that the isolated LET-23 extracellular region dimerizes with a sub-micromolar *K*_d_^dim^ in the absence of ligand. The *K*_d_^dim^ value for unliganded sLET-23 is therefore actually smaller (stronger) than *K*_d_^dim^ values measured for EGF-bound human sEGFR (∼3.3 μM^16^), which dimerizes in cell membranes. As the local concentration of EGFRs in a cell is typically in the 1 to 10μM range^16^,^19^, our results imply that LET-23 is constitutively dimerized on the cell surface through interactions driven by its extracellular region alone. However, our findings almost certainly underestimate the strength of LET-23 dimerization at the cell surface. First, we do not account for contributions to dimerization from the transmembrane, juxtamembrane or intracellular domains—which all appear to contribute to human EGFR dimerization^7^,^20^,^21^,^22^. Second, our argument does not consider pre-orientation effects that will be experienced by LET-23 in the confines of a membrane, which are estimated to further enhance dimerization by up to 10^6^-fold^23^.

Despite probably being completely dimeric in the cell membrane, LET-23 activation remains strictly dependent on addition of LIN-3 (Fig. 1e and Supplementary Fig. 2c,d). We expressed full-length LET-23 in *D. melanogaster* Schneider-2 (S2) cells as a null background and showed that LIN-3^EGF^ is sufficient to activate the heterologously expressed receptor. These first biochemical data for LIN-3/LET-23 also reveal that LIN-3, which is the only LET-23 ligand in *C. elegans*, resembles one of the low-affinity EGFR ligands in humans (amphiregulin, epigen and epiregulin^24^) or *D. melanogaster* (vein^25^). As with other known low-affinity ligands, LIN-3 shows both ED_50_ values for receptor activation (Fig. 1e and Supplementary Fig. 2c,d) and *K*_d_ values for LET-23 binding (Supplementary Fig. 2a,b) that are 10- to 100-fold higher (weaker) than typically reported for EGF or transforming growth factor-α in humans^24^ (or spitz in *D. melanogaster*^18^).

### Structural determinants of sLET-23 dimerization

**Figure 2:**
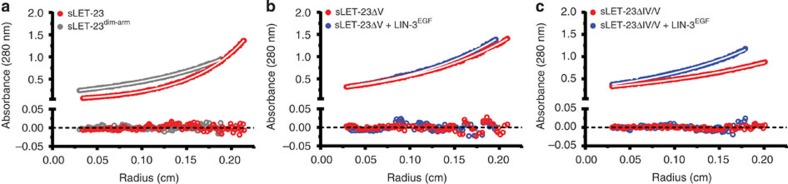
Determinants of sLET-23 dimerization. (**a**) Representative SE-AUC data for sLET-23 (red circles) and sLET-23^dim-arm^ (grey circles) collected at 5 μM and 10,000 r.p.m. (without added ligand), which fit to single ideal species of 190±22 and 94±7 kDa, respectively, across all repeats (see Table 1 and Supplementary Fig. 3). (**b**) Representative SE-AUC data for sLET-23ΔV (8 μM, 8,200 r.p.m.) with (blue circles) and without (red circles) 50 μM LIN-3^EGF^. The data fit to single species of 141±3 and 153±27 kDa with and without ligand, respectively (see Table 1 and Supplementary Fig. 3), indicating that ligand binding does not alter oligomerization state. (**c**) Representative SE-AUC data for sLET-23ΔIV/V (8 μM, 8,200 r.p.m.) with 60 μM LIN-3^EGF^ (blue circles) or without ligand (red circles). The data show that sLET-23ΔIV/V is largely monomeric (82±8 kDa), and that ligand binding promotes dimerization (see Table 1 and Supplementary Fig. 3).

Our results argue that EGFR signalling in *C. elegans* is regulated by ligand-induced structural rearrangements within receptor dimers rather than by ligand-induced changes in oligomerization, as is more commonly supposed for this receptor family. These findings also provide an opportunity to assess the structural determinants of constitutive sLET-23 dimerization and to investigate the extent and nature of the ligand-induced conformational changes. To address the first question, we mutated the so-called ‘dimerization arm’ within domain II (Fig. 1a) at six sites where analogous mutations are known to disrupt human EGFR dimerization^26^. The resulting sLET-23^dim-arm^ variant failed to dimerize in SE-AUC experiments, sedimenting as a single (monomeric) species of 94±7 kDa (Fig. 2a and Table 1). Thus, as with ligand-driven human EGFR dimers, the dimerization arm also appears important for constitutive dimerization of sLET-23. Deletion of the invertebrate-specific domain V, at the carboxy terminus of sLET-23 (Fig. 1a), did not prevent dimerization (Fig. 2b and Table 1)—consistent with results mentioned above. By contrast, deleting both domains IV and V did weaken dimerization substantially (Fig. 2c and Table 1), increasing the value of *K*_d_^dim^ from sub-micromolar to 30±13 μM. Intriguingly, this sLET-23ΔIV/V variant now displayed ligand-induced dimerization (Fig. 2c and Table 1) similar to the human or *D. melanogaster* EGFR extracellular regions, with ligand-binding strengthening dimerization by ∼6-fold (to a *K*_d_^dim^ of 5±5 μM with LIN-3^EGF^ bound).

### Ligand-induced structural changes in sLET-23

**Figure 3:**
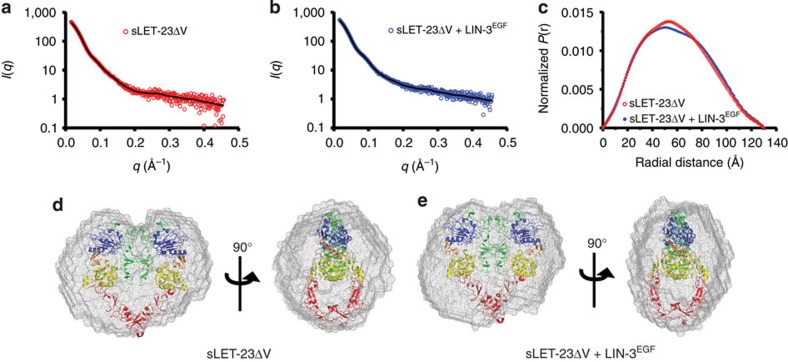
SAXS analysis of ligand-induced conformational changes in sLET-23ΔV dimers. Experimental synchrotron scattering data for 42 μM sLET-23ΔV in the absence (**a**, red circles) or presence (**b**, blue circles) of 100 μM added LIN-3^EGF^, collected at pH 8.0 at MacCHESS (see Supplementary Fig. 4). Data were acquired between 0.0164 Å^−1^≤*q*≤0.456 Å^−1^ and fit with GNOM^36^ (black line) as described in the Methods. Dilutions to 28 and 14 μM did not reveal spurious concentration-dependent effects. (**c**) SAXS-derived radial distance distributions (or *P*(r) curves) for 42 μM sLET-23ΔV with 100 μM LIN-3^EGF^ (blue points) or without added ligand (red points), calculated by inverse Fourier transforms of (**a**,**b**). SAXS-derived molecular envelopes, shown as grey mesh, for 42 μM sLET-23ΔV alone (**d**) or in the presence of 100 μM LIN-3^EGF^ (**e**). The EGF-stabilized dimer of human sEGFR from PDB entry 3NJP^4^ has been manually docked into both envelopes to illustrate the small scale of ligand-induced structural rearrangements. *D*_max_ (at 130±5 Å) is unchanged on LIN-3^EGF^ addition for these studies and the measured value for *R*_g_ is also unaltered, at 45.3±0.5 Å.

We used SAXS to monitor the extent of ligand-induced structural changes in the sLET-23ΔV dimer, following approaches that we have used previously for the human and *D. melanogaster* EGFRs^18^,^27^. Scattering curves for sLET-23ΔV in the absence and the presence of excess ligand were very similar to one another (Fig. 3a,b), although there are small differences in the region beyond *q*∼0.15 Å^−1^—suggesting ligand-induced conformational changes within the dimer that are very limited in scale. The SAXS-derived radial distance distribution or *P*(*r*) curve (Fig. 3c) for unliganded sLET-23ΔV closely resembles that calculated for the crystallographic dimer of the unliganded *D. melanogaster* EGFR extracellular region^17^ (Supplementary Fig. 4a), which is also dimerization arm-mediated. A molecular envelope derived from unliganded sLET-23ΔV SAXS data also readily accommodates a model of the EGF-induced sEGFR dimer (Fig. 3d). Thus, our SAXS data indicate that the constitutive sLET-23 dimer resembles a domain II dimerization arm-mediated dimer of the sort seen in crystal structures of the human and *D. melanogaster* EGFR extracellular regions. Adding excess LIN-3^EGF^ results in only small changes to both the *P*(*r*) curve (Fig. 3c and Supplementary Fig. 4a) and the SAXS-derived molecular envelope (Fig. 3e), arguing that there are certainly some ligand-induced conformational changes, but that they are subtle. Neither the radius of gyration (*R*_g_) nor the maximum dimension (*D*_max_) of the sLET-23ΔV dimer is altered significantly on ligand binding, remaining fixed at 45.3±0.5 and 130±5 Å, respectively (Fig. 3c). This contrasts with the ∼20% increase in both of these parameters when EGF binds to (and dimerizes) sEGFR^27^. Nonetheless, LIN-3^EGF^ binding does alter the shape of the sLET-23ΔV *P*(*r*) curve. It does so in a way that closely resembles the changes seen when comparing the *P*(*r*) curve calculated for the unliganded crystallographic dimer of the *D. melanogaster* EGFR extracellular region (in which the two molecules are ‘splayed apart’) with that for the liganded *D. melanogaster* EGFR dimer in which the two molecules are more intimately associated^17^ (Supplementary Fig. 4a). Thus, a comparison of SAXS-derived molecular envelopes for sLET-23ΔV with and without bound LIN-3^EGF^ (Fig. 3d,e) paints a picture of subtle domain rearrangements that could alter the relative positions of the membrane proximal domains within a LET-23 dimer and allow structural communication of ligand occupancy status to the intracellular tyrosine kinase domain in the full-length receptor. Although the resolution of SAXS does not allow us to define these changes precisely, a model of this sort aligns well with recent structure-based suggestions for how IR family receptors function^10^,^11^,^12^,^13^. Ligand binding to the IR dimer induces domain rearrangements that in turn alter the position of the membrane proximal domains in a manner that permits productive interactions between the intracellular tyrosine kinase domains for activation. It has been hypothesized that EGFR family members signal similarly^8^ and our data for LET-23 provide the first direct evidence for such ligand-induced structural alterations within preformed EGFR dimers.

## Discussion

Ligand binding drives dimerization and activation of many RTKs, including the human and *D. melanogaster* EGFRs^1^,^17^, although it seems clear that these receptors dimerize to some extent even in the absence of ligand^8^. The IR paradigm, and now our data with the *C. elegans* EGFR LET-23, argue that although RTK dimerization is necessary for activation, it is not sufficient and need not necessarily be ligand induced. A ligand-induced dimerization step in the activation of an RTK may provide an additional level of regulation, and one that is exploited by the human EGFR but not LET-23 in *C. elegans*. Indeed, certain phosphorylation sites such as threonine 654 in the intracellular juxtamembrane region of human EGFR that have regulatory roles^28^, and may modulate dimerization, are absent in LET-23. Moreover, as in several other RTK families, the single EGFR of *C. elegans* (or *D. melanogaster*) is replaced by several orthologues in mammals—allowing signal diversification through receptor heterodimerization^29^. Once dimers are formed, however, the results described here and current models for the IR argue that a precise conformation is required for RTK activation. By belonging to a class of RTKs typically associated with ligand-induced dimerization, yet signalling as a constitutive dimer (without ligand-induced changes in oligomerization state), the *C. elegans* EGFR, LET-23, appears to represent a ‘missing link’ between the IR family and other RTKs^9^. Understanding the structural transitions in more detail, and how they are translated into intracellular kinase domain activation, will be a key step in appreciating the significance of the much discussed preformed dimers of EGFRs and other RTKs.

## Methods

### Protein expression and purification

Coding regions for sLET-23 variants were subcloned with a C-terminal hexahistidine tag into pFastbac-1 (Invitrogen) for expression in *Spodoptera frugiperda* (Sf9) cells. Constructs included the native signal sequence and ended at M819 (sLET-23), K645 (sLET-23ΔV) or E539 (sLET-23ΔIV/V). ‘Dimerization arm’ mutations made in domain II of sLET-23 were modelled on those shown to abolish sEGFR dimerization^26^ (F301D, N302A, K304D, G306E, R307A and L308D), and were generated using QuikChange (Stratagene).

DNA encoding the LIN-3 EGF domain (LIN-3^EGF^: residues K148-N206, with numbering from isoform F36H1.4c on Wormbase) was subcloned into a pMT-V5-His (Invitrogen) variant for expression in *D. melanogaster* S2 cells as described^30^. The expressed protein contains a BiP signal sequence, followed by a hexahistidine tag, residues 44–76 of *D. melanogaster* Spitz, and then (after a Factor Xa cleavage site) LIN-3^EGF^, which is released from this fusion protein by Factor Xa cleavage. For expressing full-length LIN-3 (LIN-3^FL^), DNA encoding its mature extracellular region (isoform F36H1.4c on Wormbase) was subcloned into pFastbac-1 for Sf9 cell expression, with native signal sequence, a C-terminal hexahistidine tag (following residue S215) and a C23S mutation to prevent possible amino-terminal acylation^31^.

Stable S2 cell pools and recombinant baculoviruses were produced as described^17^. sLET-23 and LIN-3^FL^ were purified from the medium of Sf9 cells that had been infected with baculovirus at a density of 1.5–2.5 × 10^6^ cells per ml. Conditioned medium was harvested 3–4 days post infection and flowed over a Ni-NTA (Qiagen) affinity column. The column was washed with 25 mM MES, pH 6.0, 150 mM NaCl (buffer A) and bound protein eluted with an increasing gradient of imidazole in buffer A. For sLET-23, protein-containing fractions were concentrated with an Amicon Ultra 30K concentrator (Millipore), exchanged into buffer A to remove imidazole, and loaded onto an SO_3_^−^ cation exchange column that was developed using a gradient from 150 mM to 1 M NaCl in buffer A. Fractions containing sLET-23 were pooled, concentrated and applied to a Superose 6 10/300 GL column (GE Healthcare) equilibrated in 10 mM Hepes, pH 8.0, 150 mM NaCl (buffer B). The final purity of sLET-23 was >95% by Coomassie-stained SDS–PAGE. Following Ni-NTA affinity chromatography, LIN-3^FL^ was concentrated and purified further using a Superose 12 column (GE Healthcare) equilibrated in buffer B. Human sEGFR was produced exactly as described^32^.

LIN-3^EGF^ was purified from 500 ml of S2 cells grown to ∼4–6 × 10^6^ cells per ml and induced with 750 μM CuSO_4_ for 4 days. Conditioned medium was dialysed in 24 l of 10 mM Hepes, pH 8.0, 100 mM NaCl for 16 h at 4 °C and then bound to a 2-ml Ni-NTA column. After washing with buffer A, bound protein was eluted with an increasing gradient of imidazole in buffer A. LIN-3^EGF^ was concentrated, exchanged into buffer A to remove imidazole, and purified by cation exchange as for sLET-23. LIN-3^EGF^ was then exchanged into 10 mM Hepes, pH 7.0, 100 mM NaCl, 2 mM CaCl_2_, concentrated to 1 mg ml^−1^ and cleaved overnight at 4 °C with 20 μg Factor Xa. Cleaved protein was then applied to a Ni-NTA column to remove the His-tagged N terminus and the LIN-3^EGF^-containing flow-through was concentrated and further purified by size exclusion using a Superdex peptide column (GE Healthcare) equilibrated in buffer B.

### Size exclusion and MALS experiments

Size-exclusion chromatography experiments employed a Superose 6 10/300 GL column (GE Healthcare) equilibrated in buffer B. The column was calibrated with thyroglobulin (670 kDa), γ-globulin (158 kDa), ovalbumin (44 kDa), myoglobin (17 kDa) and vitamin B_12_ (1.4 kDa). Experimental proteins were loaded at 4 mg ml^−1^ in buffer B and molecular masses were determined using a DAWN HELEOS II MALS with an on-line Optilab T-rEX interferometric refractometer (Wyatt Technology). The refractive index increment (d*n*/dc) was estimated as 0.185 and the molecular mass of each protein within defined chromatographic peaks was calculated using ASTRA software version 6.0 (Wyatt Technology) as recommended by the manufacturer.

### Sedimentation equilibrium analytical ultracentrifugation

SE-AUC experiments employed an XL-A analytical ultracentrifuge with an An-60 Ti rotor (Beckman Coulter, Fullerton, CA). Samples of sLET-23 proteins ranging from 1 to 8 μM in buffer B were analysed with and without addition of 50–70 μM LIN-3^EGF^ in six-channel charcoal-Epon cells as described previously for our studies of human EGFR^33^, at 20 °C, at three speeds (between 5,000 and 12,500 r.p.m.), detecting at 280, 290 and 295 nm. Radial positions are plotted in figures as *r*−*r*_0_, where *r*_0_ is the meniscus. The partial specific volume of sLET-23 was estimated as 0.71 ml g^−1^, solvent density as 1.003 g ml^−1^ and buffer viscosity as 1.0204 × 10^−2^ cPoise. For each sample, equilibrium data at three speeds were analysed as a group with HeteroAnalysis (v1.1.57, written by J. Cole and J. Lary, University of Connecticut) and were fit as a single ideal species. To estimate *K*_d_^dim^ for sLET-23ΔIV/V, data from three concentrations were analysed as a group and fit in HeteroAnalysis to a model describing simple receptor dimerization, floating only *K*_d_^dim^. Errors quoted in the text are means±s.d. for 3–18 independent measurements.

### Small angle X-ray scattering

Home-source data were recorded at 4 °C on a Rigaku PSAXS S-Max3000 pinhole camera system with a Rigaku 007HF rotating anode source and a Rigaku 300 mm wire grid ASM DTR 200 detector, with 20–80 min exposures. Protein concentration ranged from 1.2 to 5 mg ml^−1^ in buffer B (pH 8.0). EGF was added to sEGFR samples at a 1.2-fold molar excess and LIN-3^EGF^ was added in larger excesses (adding 61–110 μM), to reach >90% saturation of sLET-23ΔV. In the latter case, LIN-3^EGF^ at the concentration estimated to be unbound (based on the *K*_d_ values reported in Supplementary Fig. 2) was included in the buffer blank for subtraction. Home-source data were reduced using SAXSGui v2.05.02 (Rigaku America & JJ X-Ray Systems ApS, Lyngby, Denmark) and matching buffers were subtracted using PRIMUS^34^, to yield the final scattering profile in which intensity (*I*) is plotted as a function of *q* (4*π*sin*θ*/*λ*, where 2*θ* is the scattering angle). Synchrotron data were acquired at CHESS beamline F1, operating at 12.686 keV. Samples were protected from radiation damage by the addition of 5% v/v glycerol to buffer B and oscillation in the X-ray beam using a computer-controlled syringe pump. The X-ray beam was collimated to 250 × 250 μm^2^ and images were collected with a Dectris Pilatus 100 K-S detector, with sample-to-detector distance of 1,140 mm and momentum transfer interval of 0.0164 Å^−1^<*q*<0.456 Å^−1^. Ten exposures of 4 s were averaged and the data were subtracted and reduced using BioXTAS RAW software^35^.

All samples were monodisperse as evidenced by linear Guinier regions where *qR*_g_≤1.3 and Kratky plots showed no signs of radiation-induced denaturation. *P*(*r*) curves and values for *I*(0), *R*_g_ and *D*_max_ were obtained using the programme GNOM^36^. *R*_g_ values calculated with GNOM were consistent with those estimated from the Guinier region, and the total estimate for each GNOM run was better than 0.90. *I*(0)/c measurements (Fig. 1d) were all performed using our home source, taking advantage of beam stability. Measured intensities were converted to absolute units during data reduction using beam transmission corrections and the scattering from glassy carbon. *I*(0) values were normalized by mass concentration, and parallel measurements using reference proteins of known mass were consistent with literature values^37^: lysozyme (14.6 kDa), ovalbumin (44.3 kDa) and glucose isomerase (173 kDa). The structural information described in Fig. 3 are all from data collected at CHESS. Using synchrotron scattering data up to *q*_max_=0.3 Å^−1^, low-resolution molecular envelopes (Fig. 3d,e) were constructed by averaging 20 independent DAMMIN^38^ runs (with no imposed symmetry) using the programme DAMAVER^39^. Mean normalized spatial discrepancy values for sLET-23ΔV and sLET-23ΔV with bound LIN-3^EGF^ were 0.76 and 0.77, respectively. pH-dependent differences in SAXS parameters seen for unliganded sLET-23ΔV in our home-source experiments (see Supplementary Fig. 4b,c) were not observed in experiments performed at CHESS. Molecular mass estimates computed at CHESS for sLET-23ΔV (derived from comparison of *I*(0)/c with lysozyme and glucose isomerase standards) were also consistent with constitutive sLET-23ΔV dimerization.

### Analysis of LET-23 activation

*D. melanogaster* S2 cells were chosen as a null background, because no *D. melanogaster* EGFR activation can be detected when these cells are treated with its activating ligands^40^ and, in addition, we cannot detect binding of LIN-3 to the *D. melanogaster* EGFR extracellular region. S2 cells stably transfected with full-length LET-23 (containing a C-terminal hexahistidine tag) were serum starved overnight. LET-23 expression was induced for 3.5 h with 60 μM CuSO_4_ and LIN-3^EGF^ was added at the indicated concentrations for 10 min on ice, in PBS containing 0.5% (w/v) BSA. Cells were then lysed in ice-cold 25 mM Tris, pH 7.5, 150 mM NaCl, 1% Nonidet P40 containing protease and phosphatase inhibitors. His-tagged LET-23 was enriched from clarified lysates by Ni-NTA affinity precipitation and samples were analysed by western blotting using phosphotyrosine (α-pY20; Santa Cruz Biotechnology) and His_5_ (Qiagen) antibodies at dilutions of 1:500 and 1:1,000, respectively, followed by incubation with relevant secondary antibodies conjugated to fluorescent dyes (LI-COR Biosciences, Lincoln, NE) for detection at 680 and 800 nm using LI-COR Odyssey imaging.

### Surface plasmon resonance

Surface plasmon resonance experiments employed a Biacore 3,000 instrument. LIN-3^FL^ and LIN-3^EGF^ were immobilized on a Biacore CM5 biosensor chip using amine coupling as described^32^. Purified sLET-23 was injected at the indicated concentrations at 5 μl min^−1^ for 9 min (sufficient for binding to reach steady state), in degassed 10 mM Hepes (pH 8.0), 150 mM NaCl, 3 mM EDTA and 0.005% Surfactant P-20 at room temperature. The final steady-state signal was background corrected by subtraction of the signal obtained with the control surface. Values were plotted against [sLET-23 variant] and fit to a simple single-site saturation-binding model. Between injections, the surface was regenerated using a 10-μl injection of 10 mM sodium acetate, pH 5.0, 1 M NaCl.

## Additional information

**How to cite this article**: Freed, D. M. *et al*. Ligand regulation of a constitutively dimeric EGF receptor. *Nat. Commun.* 6:7380 doi: 10.1038/ncomms8380 (2015).

## Supplementary Material

Supplementary Information Supplementary Figures 1-4 and Supplementary References

## Figures and Tables

**Table 1:** Molecular masses from SE-AUC fits.

**Receptor**	**Mass**^**pred**^ **(kDa)**	**Mass**^**fit**^ **(kDa)**
sLET-23	91	190±22
sLET-23+LIN-3^EGF^	98	185±6
sLET-23ΔV	71	153±27
sLET-23ΔV+LIN-3^EGF^	78	141±3
sLET-23ΔIV/V	60	82±8
sLET-23ΔIV/V+LIN-3^EGF^	67	123±8
sLET-23^dim-arm^	91	94±7

SE-AUC, sedimentation equilibrium analytical ultracentrifugation.

Mass^pred^ is the polypeptide molecular mass of the monomeric protein, as predicted from the primary sequence. Mass^fit^ corresponds to the molecular mass obtained by fitting the experimental data at three speeds for each variant to a single species (for which residuals were small and random). Average Mass^fit^ values at a concentration of 8 μM are shown for sLET-23ΔIV/V; all other receptor variants were fully dimeric (or monomeric in the case of sLET-23^dim-arm^) at all concentrations tested (from 1 to 8 μM) and Mass^fit^ values are quoted as the concentration-independent average (±s.d.). sLET-23 contains six predicted N-glycosylation sites compared with nine for human sEGFR (which is ∼20% carbohydrate by mass and ∼71 kDa polypeptide). We therefore estimate that sLET-23 is ∼10% carbohydrate by mass. Estimates for sLET-23ΔV and sLET-23ΔIV/V are ∼9% (4 sites) and ∼11% (4 sites), respectively. See Supplementary Fig. 3 for representative data fits.
