# Supplementary material for: Ligand regulation of a constitutively dimeric EGF receptor
Source: Nat Commun. 2015 Jun 10;6:7380. doi: 10.1038/ncomms8380 (PMC4465127; doi:10.1038/ncomms8380)
Supplement: Supplementary Information — Supplementary Figures 1-4 and Supplementary References [file ncomms8380-s1.pdf]

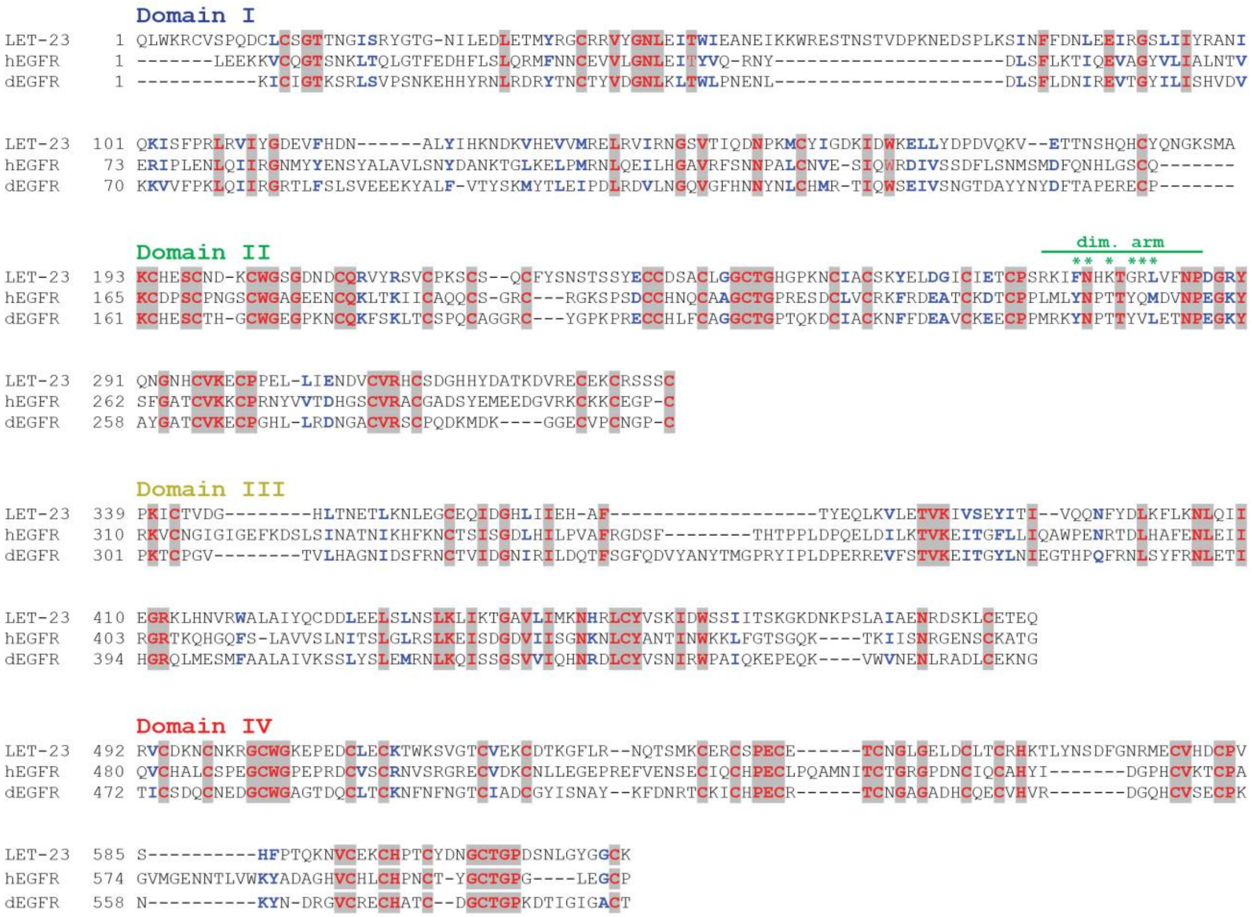

Supplementary Figure 1 | Sequence alignment of domains I-IV in the extracellular regions of LET-23, human EGFR (hEGFR), and *D. melanogaster* EGFR (dEGFR). Generated using Clustal Omega and BOXSHADE. Regions of sequence identity are highlighted in grey, and are represented by bold red amino acid identifiers. Areas of sequence similarity are represented by bold blue amino acid identifiers. The green line above the middle of the domain II sequence represents the dimerization arm, and sites where mutations were made to create the sLET-23$^{\text{dim-arm}}$ construct are shown as green asterisks. Amino acid numbering is for the predicted mature proteins (minus signal sequence).

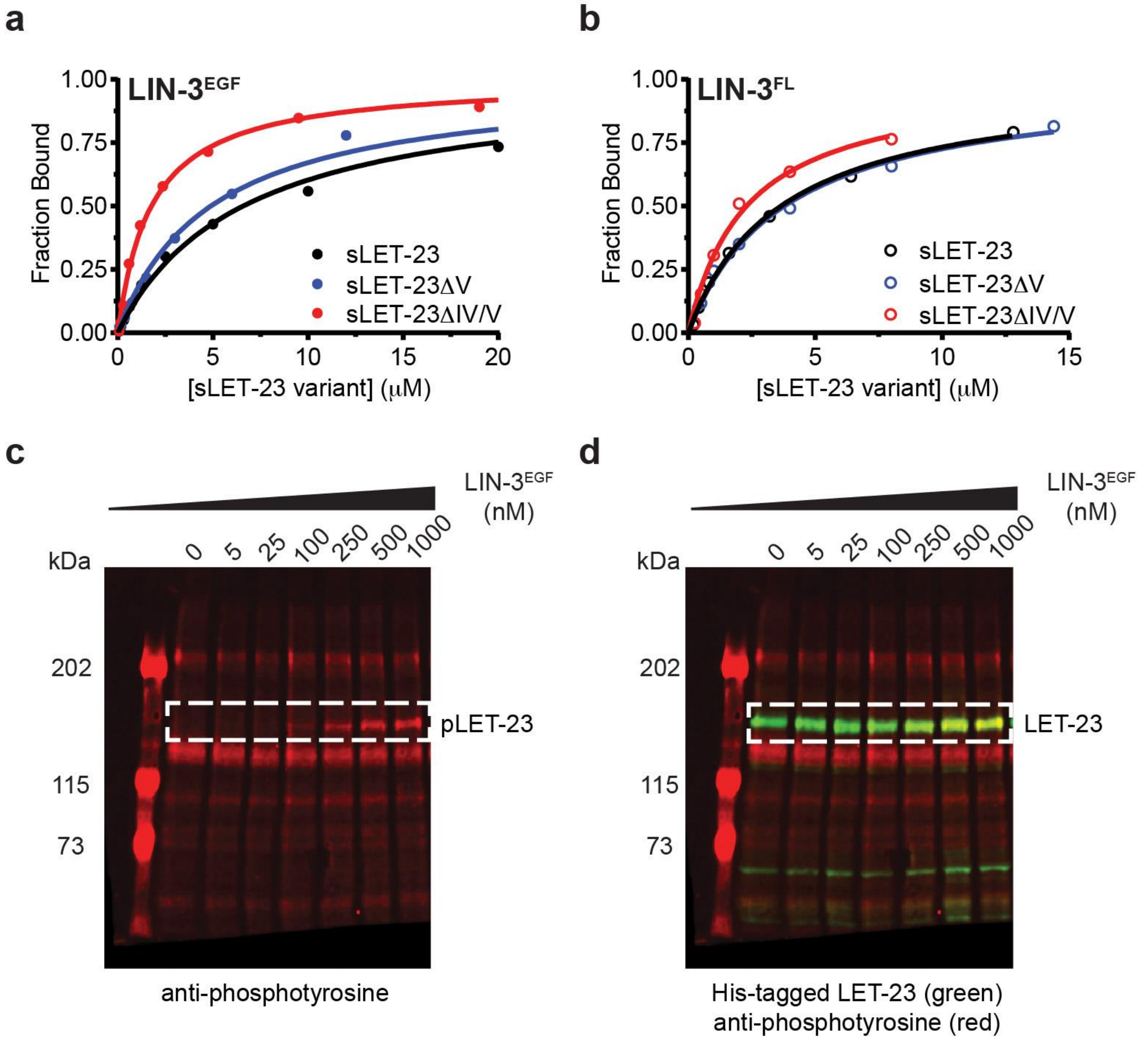

Supplementary Figure 2 | LIN-3 binding to LET-23. (**a,b**) Binding of sLET-23 variants to LIN-3^{EGF} (**a**) and full-length LIN-3 (LIN-3^{FL}) (**b**) immobilized on CM-5 sensorchips, monitored using SPR. All values for K_d (for over 8 experiments) fell between 1.8 μM and 6.6 μM, regardless of whether the ligand was LIN-3^{EGF} or LIN-3^{FL}, and whether or not sLET-23 contained domains IV and V. K_d values were estimated by fitting the binding curves to a simple single-site hyperbolic binding equation in GraphPad Prism. (**c**) Complete gel for the LI-COR Western blot shown in Fig. 1e, illustrating LIN-3^{EGF}-induced LET-23 autophosphorylation in S2 cells. The cropped region shown in Fig. 1e is outlined by a white dashed line. The phosphoLET-23 bands migrate near their predicted molecular weight of approximately 150 kDa. (**d**) Complete two-color (LI-COR) Western blot of LET-23 activation. The cropped region shown in Fig. 1e is again outlined by a white dashed line. The His_5 channel (denoting His-tagged, full-length LET-23 levels) is shown in green and the pY20 channel (denoting phospho-tyrosine levels) is shown in red.

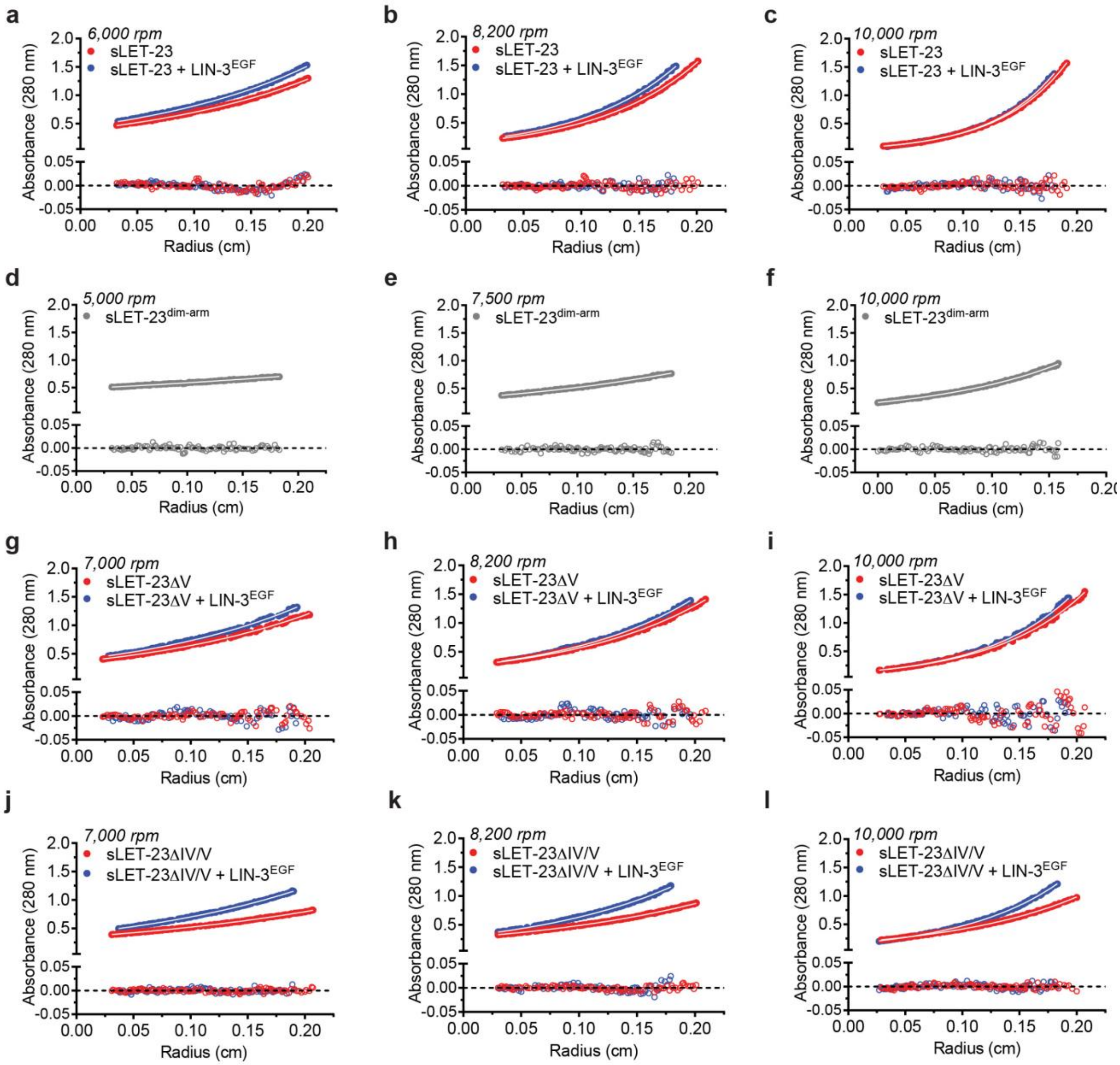

Supplementary Figure 3 | SE-AUC data used for fitting the molecular masses of sLET-23 variants. Constructs are described in Fig. 1c and Fig. 2. For each sample, the data at three rotor speeds were fit as a group to a single ideal-species model. The fits are shown as white lines through the data, with the residuals shown below. For data of sLET-23 with bound LIN-3^{EGF}, signal arising from excess ligand was subtracted as background during the fitting process. Molecular masses from these fits are reported in the text and in Table 1. Where quoted, global fits for the sLET-23 variant at three concentrations were used to estimate K_d^{dim} values.

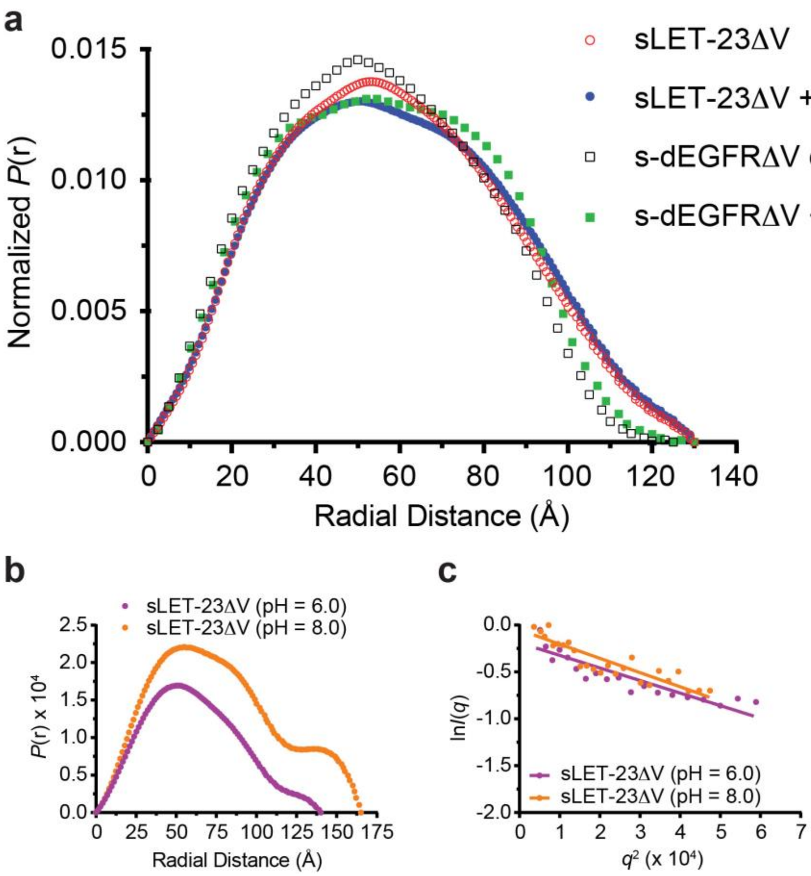

Supplementary Figure 4 | SAXS studies of sLET-23ΔV. (**a**) Radial distance distribution (P(r)) curves for sLET-23ΔV from MacCHESS data with (blue circles) and without (red circles) bound LIN-3^{EGF} are overlaid with those for the crystallographic s-dEGFRΔV dimer with- (green squares) and without (open black squares) bound Spitz^{EGF}. Experimental sLET-23ΔV P(r) curves were generated using GNOM[1] from the fits shown in Fig. 3a,b. Theoretical P(r) curves for s-dEGFRΔV were created from crystal-structure-based models using CRYSOL[2] and GNOM[1]. The s-dEGFRΔV dimer seen in PDB entry 3I2T[3] was used for the unliganded dimer, and PDB entry 3LTF[4] was used for the Spitz^{EGF}-bound s-dEGFRΔV dimer. In both cases, domain IV was completed by modeling based on other ErbB structures. Data are normalized by area under the P(r) curve. This comparison of P(r) curves reveals that the nature and extent of ligand-induced conformational changes in the sLET-23ΔV dimer are similar in scale to those seen in s-dEGFRΔV dimers upon Spitz^{EGF} binding. (**b**) We observed some pH-dependent differences in sLET-23ΔV scattering in home-source experiments at high protein concentrations and relatively long exposure times. In buffer B at pH 8.0 (orange circles), the average normalized I(0)/c for sLET-23ΔV was 2.2 times larger than for monomeric human sEGFR. However, in 25 mM MES, 150 mM NaCl at pH 6.0 (purple circles), I(0)/c is reduced to 1.5 – closer to the expected value for sLET-23ΔV dimers given the reduced glycosylation compared with human sEGFR, and similar to the I(0)/c value measured for sLET-23ΔV plus LIN-3^{EGF} at pH 8.0 with the home source (note that ligand binds very weakly at pH 6.0). Moreover, R_g and D_{max} for unliganded sLET-23ΔV were 55.6 ± 3.4 Å and 155 Å respectively at pH 8.0, but just 48.0 Å and 140 Å respectively at pH 6.0. The latter numbers agree well with those obtained at CHESS (Fig. 3) at pH 8.0 (R_g = 45.3 ± 0.5 Å, D_{max} = 130 Å) with shorter exposures and lower protein concentrations. Since LIN-3^{EGF}-bound sLET-23 always had dimensions equal to or smaller than dimer (or those of unliganded sLET-23) these results further underscore the basic conclusion that unliganded sLET-23 oligomerizes, and that oligomerization is not enhanced by ligand binding. (**c**) Guinier analysis shows that the pH-dependent differences in SAXS-derived parameters do not arise from protein aggregation. Guinier regions ($q*R_g$ < 1.3) for sLET-23ΔV at both pH = 6.0 (purple circles) and at pH = 8.0 (orange circles) are linear.

Supplementary References

1. Semenyuk, A. V. & Svergun, D. I. Gnom - a Program Package for Small-Angle Scattering Data-Processing. *J. Appl. Crystallogr.* **24**, 537-540 (1991).
2. Svergun, D. I., Barberato, C. & Koch, M. H. J. CRYSOL - a program to evaluate X-ray solution scattering of biological macromolecules from atomic coordinates. *J. Appl. Cryst.* **28**, 768-773 (1995).
3. Alvarado, D., Klein, D. E. & Lemmon, M. A. ErbB2 resembles an autoinhibited invertebrate epidermal growth factor receptor. *Nature* **461**, 287-291 (2009).
4. Alvarado, D., Klein, D. E. & Lemmon, M. A. Structural basis for negative cooperativity in growth factor binding to an EGF receptor. *Cell* **142**, 568-579 (2010).
